# Regulation of Matrix Metalloproteinases by Wine-Derived Compounds: Implications for Cancer Therapy

**DOI:** 10.3390/biom15060781

**Published:** 2025-05-28

**Authors:** Md. Towhedul Islam, Ha Vy Thi Vo, Hyuck Jin Lee

**Affiliations:** Department of Chemistry Education, Kongju National University, Gongju-si 32588, Chungcheongnam-do, Republic of Korea; towhedchem15@gmail.com (M.T.I.); vothihavy11102000@gmail.com (H.V.T.V.)

**Keywords:** wine, flavonoids, metalloenzymes, matrix metalloproteinase (MMP), cancer

## Abstract

Cancer remains a prevalent global health concern, with key factors such as diet, environment, and genetics playing significant roles in its progression. Matrix metalloproteinases (MMPs), particularly MMP-2 and MMP-9, play a critical role in cancer progression by degrading the extracellular matrix, thereby facilitating tumor growth and metastasis. Wine contains various bioactive compounds, including caffeic acid, gallic acid, kaempferol, naringenin, quercetin, myricetin, resveratrol, epigallocatechin gallate, riboflavin, and folic acid, which have shown promise in inhibiting cancer cell proliferation and metastasis. These compounds have been reported to downregulate the activity and/or expression of MMP-2 and MMP-9, thus potentially suppressing tumor progression. However, excessive alcohol consumption can lead to addiction and elevate the risk of various health complications, including fatty liver disease, cardiovascular damage, stroke, and kidney failure. Despite these concerns, moderate wine consumption has been associated with potential anticancer properties by restricting tumor growth and metastasis. In this review, we summarize and discuss how bioactive molecules in wine regulate MMP-2 and MMP-9 through bioactive compounds derived from wine and explore their implications for cancer treatment.

## 1. Introduction

Alcohol consumption, including wine, has been implicated in nearly 4% of global cancer cases, with even moderate intake levels significantly contributing to cancer risk [1]. In 2020 alone, more than 100,000 new cancer cases were attributed to minimal alcohol consumption. Various types of cancer, including esophageal (189,700 cases), liver (154,700 cases), breast (98,300 cases), colorectal (91,500 cases), oral cavity (74,900 cases), rectal (65,100 cases), pharyngeal (39,400 cases), and laryngeal (27,600 cases) cancers, have been linked to alcohol consumption [1,2]. The main organic component of alcoholic beverages is ethanol, which contributes to tumor development through various mechanisms. These mechanisms include the inhibition of DNA methylation, promotion of lipid peroxidation, and activation of pro-carcinogens, ultimately leading to the generation of prostaglandins, reactive oxygen species (ROS), and other polar compounds that facilitate cancer progression [3,4,5]. Therefore, a promising approach to counteract cancer development is chemoprevention, which involves the use of natural or synthetic compounds to inhibit, delay, or reverse tumorigenesis [6].

However, the health effects of ethanol in wine remain controversial. While ethanol has been shown to increase high-density lipoprotein (HDL) cholesterol, inhibit platelet aggregation, and promote fibrinolysis, potentially offering cardiovascular benefits [7], its overall impact on mortality is complex. Some meta-analyses suggest that moderate wine consumption (0–30 g/day of alcohol) can reduce overall mortality by nearly 15% [8]. In cancer patients, long-term moderate wine consumption has been associated with a 26% lower risk of secondary malignancies or recurrence, and a 33% reduction in cancer-related mortality within five years of diagnosis [9]. Furthermore, in wine, ethanol itself may play a facilitating role in enhancing the absorption and systemic bioavailability of bioactive compounds. As a solvent, ethanol can increase the intestinal permeability of polyphenols and other secondary metabolites, thereby potentially amplifying their therapeutic effects [10,11]. Furthermore, individuals who consume moderate amounts of wine (i.e., a glass of wine daily) appear to have a lower risk of Barrett’s esophagus, a precursor to esophageal adenocarcinoma, compared to heavy drinkers or abstainers [12].

Wine contains a diverse range of bioactive compounds, including flavonoids (i.e., quercetin, kaempferol, myricetin, luteolin, apigenin, epicatechin, epigallocatechin gallate, taxifolin, genistein, naringenin, naringin, hesperetin, xanthohumol, isoxanthohumol) and non-flavonoids (i.e., gallic acid, ellagic acid, *p*-coumaric acid, piceatannol, resveratrol, riboflavin, folic acid), as summarized in Table 1 [13]. Importantly, the composition of these bioactive compounds differs significantly across wine types. Red wines, due to extended fermentation with grape skins, generally contain higher levels of polyphenols such as resveratrol, quercetin, and catechins [14]. In contrast, white wines, which are fermented without skins, tend to have lower concentrations of these polyphenols but may contain other compounds such as tyrosol and hydroxytyrosol [14,15]. Dry wines contain minimal residual sugar and tend to preserve higher levels of polyphenols and organic acids, while sweet wines, especially those from late-harvest or dried grapes, often show elevated sugar levels and modified phenolic compositions [16,17]. These compositional differences may influence both the bioavailability and biological activity of wine-derived bioactive compounds. Notably, these compounds possess antioxidant properties and modulate cancer cell behavior by regulating various intracellular signaling pathways [13,18]. Moreover, they can interfere with the cell cycle, induce apoptosis, and inhibit angiogenesis, thereby suppressing tumor progression [17,18,19].

Matrix metalloproteinases (MMPs) are a family of zinc-dependent endopeptidases that play a critical role in extracellular matrix (ECM) remodeling [33]. MMPs regulate key ECM components, including collagen, elastin, and gelatin, which are essential for tissue homeostasis, wound healing, and various pathological processes [34]. Structurally, MMPs typically consist of a catalytic domain, an N-terminal pro-domain, a hinge region, and a C-terminal hemopexin-like domain [35]. Dysregulation of MMP activity has been implicated in multiple diseases, including cancer, autoimmune disorders, cardiovascular diseases, and chronic inflammation [36,37]. Among the MMP family, MMP-2 and MMP-9 are particularly significant in cancer progression [38]. These enzymes degrade ECM components, including Type IV collagen, a fundamental component of basement membranes, thereby facilitating cancer cell invasion into surrounding tissues and blood vessels. This degradation enables tumor cells to enter the circulation and metastasize to distant organs [23,39].

In addition, MMP-2 and MMP-9 promote tumor progression by modulating the tumor microenvironment through the activation of chemokines and proinflammatory cytokines, which recruit immune cells that may support tumor growth and survival [36,40]. Furthermore, these enzymes contribute to tumor vascularization, supplying oxygen and nutrients to cancer cells and enabling metastasis, the primary cause of cancer-related mortality [39,41,42,43,44,45,46]. Due to the crucial role of MMP-2 and MMP-9 in cancer progression, targeting their regulation has emerged as a promising therapeutic approach [38,44].

Chronic alcohol consumption has been known to be associated with a higher incidence of multiple malignancies, including cancers of the rectum, female breast, pharynx, larynx, liver, esophagus, colon, and oral cavity [46,47]. However, bioactive components in wine may exert protective effects by modulating MMP activity and expression, thereby influencing cancer progression [13]. In this review, understanding how the bioactive compounds in wine influence MMPs, specifically MMP-2 and MMP-9, provides valuable insights into their potential implications for cancer therapy.

While several previous reviews have addressed the modulation of MMPs by bioactive compounds such as natural compounds, this work provides an updated synthesis of recent findings about bioactive compounds in wine, highlighting novel mechanisms and therapeutic potentials reported in the latest literature.

## 2. Role of Wine-Derived Compounds for MMP Regulation in Cancer Pathology

### 2.1. Progress of Metastasis by ECM Degradation

MMP-2 (gelatinase A) and MMP-9 (gelatinase B) are the two main components of the gelatinase family [48,49]. The signal peptide, propeptide, hemopexin-like domain, and catalytic domain with a zinc-binding site are all present in MMP-2 and MMP-9. The three fibronectin type II-like domains that adhere to the catalytic domain make it feasible to bind and degrade collagen types IV, V, VII, X, and ECM [50].

**Figure 1:**
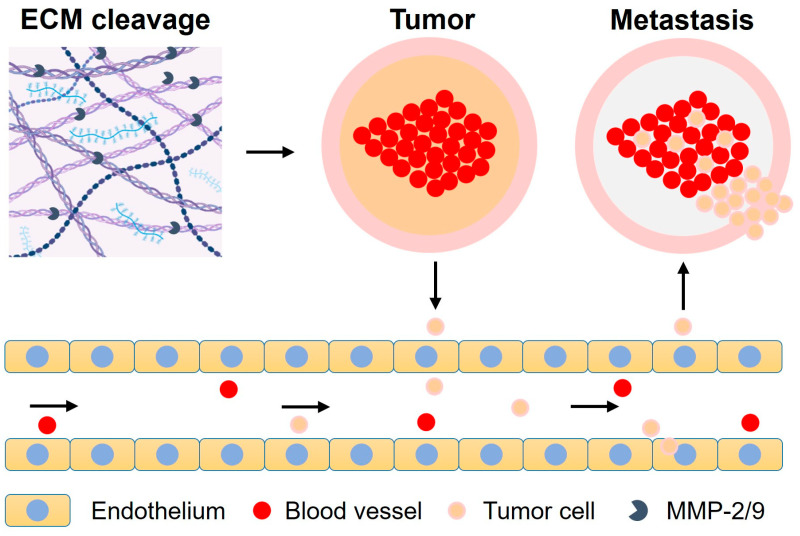
Progress of metastasis by MMP-2 and MMP-9.

Many physiological processes, such as tissue remodeling and repair, cellular differentiation, cell migration and proliferation, angiogenesis, wound healing, and apoptosis, depend on MMP activity or expression [51]. However, a number of clinical illnesses, including arthritis, neoangiogenesis, atherosclerosis, cardiovascular diseases, neurological diseases like Alzheimer’s and Parkinson’s diseases, and different types of cancer, have been reported to involve MMP-mediated ECM degradation [52]. Tumor angiogenic processes are significantly regulated by interactions between tumor cells, endothelial cells, and the extracellular matrix. Together with the hypoxia and nutritional deprivation that induce tumor formation, tumor vascularization mechanisms produce the circumstances necessary for long-term tumor proliferation and enable tumor cells to move from their initial spot to detached metastatic sites summarized in Figure 1 [53,54].

Apoptosis, growth, differentiation, migration, metastasis, invasion, and resistance to treatment are all impacted by abnormalities in the PI3K/AKT pathway, which are commonly seen in several malignancies [55]. In addition, NF-κB dysregulation leads to tumor evolution and active NF-κB can be detected in a variety of malignancies. Tumor development and metastasis are caused by elevated NF-κB gene expression [56]. While cancer is caused by various signaling pathways, we concentrated on controlling their activity. Many key biochemical pathways are known to be involved in the regulation

### 2.2. Wine Compounds as Signaling Pathway Inhibitors

**Figure 2:**
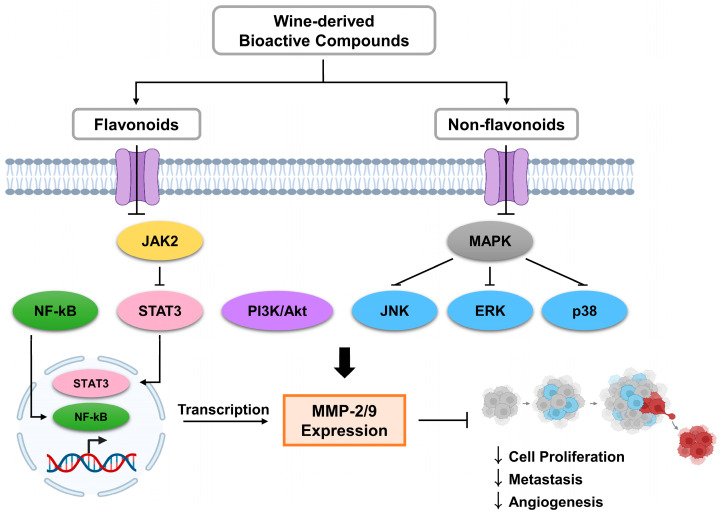
Mechanism of MMP-2 and MMP-9 regulation by wine-derived compounds.

Many key biochemical pathways are known to be involved in the regulation of MMPs in cancer pathophysiology, thereby facilitating tumor invasion and migration as shown in Figure 2. Among them, the MAPK, NF-κB, PI3K/Akt, and JAK/STAT pathways are frequently activated in malignancies and are responsible for cancer cell survival [57,58]. Bioactive compounds found in wine, including both flavonoids and non-flavonoids, have demonstrated the ability to modulate these signaling cascades, leading to the influence of cancer cell proliferation, survival, and apoptosis as presented in Figure 2. For example, quercetin and resveratrol modulate key signaling pathways such as MAPK and PI3K/Akt, consequently affecting cancer cell growth and proliferation [59]. Several polyphenols also suppress NF-κB activation, resulting in decreased transcription of pro-inflammatory cytokines and MMPs, particularly MMP-2 and MMP-9 [60]. In addition, compounds like luteolin and ellagic acid have been reported to downregulate the JAK2/STAT3 pathway, limiting cancer cell immune evasion and growth [61,62]. The PI3K/Akt pathway, a central mediator of angiogenesis and cell survival, can also be inhibited by various wine-derived flavonoids, reducing metastatic potential [63].

### 2.3. Anti-Cancer Effects of Wine

**Figure 3:**
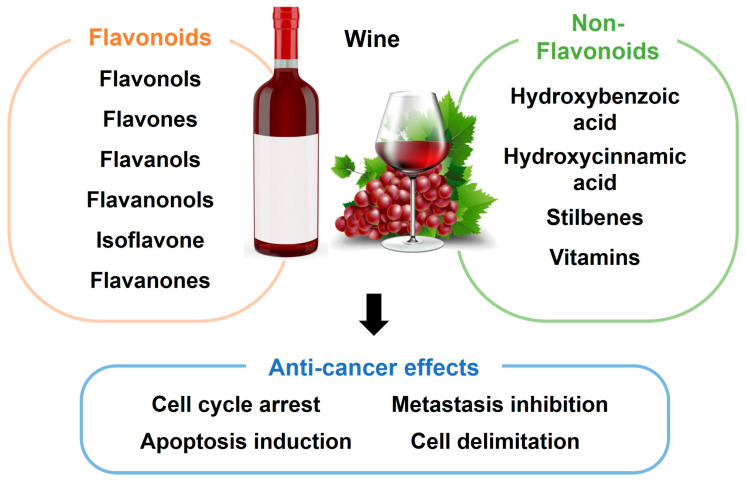
Different anti-carcinogenic activities are promoted by bioactive compounds in wine.

Flavonoids and non-flavonoid phenolic compounds are bioactive components naturally present in grape wine [64]. These compounds exert anti-cancer effects by modulating key cellular processes, including cell cycle arrest [64], apoptosis [65], metastasis inhibition [66], and cell delimitation (Figure 3) [67]. The ECM plays a crucial role in cancer progression, and its degradation is mediated mainly by urokinase-type plasminogen activator (uPA) and MMPs. A study by Dinicola et al. demonstrated that grape seed extract (GSE), which contains catechins, can inhibit breast cancer cell growth by targeting these enzymes [64,68]. Specifically, low doses of GSE (25 mg/mL) significantly suppressed MDA-MB-231 cell invasion and migration by downregulating the expression of NF-kB, fascin, b-catenin, uPA, MMP-2, and MMP-9. In contrast, higher doses of GSE (50 and 100 mg/mL) induced cell cycle arrest and apoptosis [68].

## 3. Bioactive Compounds in Wine as Regulators of MMP-2 and MMP-9

Although MMP expression is normally rather modest, elevated MMP levels have been observed in several cancer types and are linked to enhanced tumor growth and proliferation.

In normal and disease conditions, MMP-2 and MMP-9 play crucial processes of extracellular matrix remodeling. Certain ECM constituents, including laminin, collagen, and fibrin, are completely or partially damaged by cancer cells during this remodeling [69]. Analysis of MMP-2 and MMP-9 levels in tissues from radical prostatectomy demonstrated that these metalloproteinases were significant warning signs of cancer recurrence. The development, spread, and metastasis of breast, lung, colon, and stomach cancers are all greatly affected by MMP-9. Elevated levels of MMP-2 and MMP-9 have been implicated in the progression and metastasis of various cancers, including those of the bladder, breast, cervix, colon, and others [70].

It has been demonstrated that the intragenic hypermethylation of the MMP-9 gene in melanoma is linked to MMP-9 expression. Controlling MMP-2/9 expression helps preserve tissue integrity by preventing the excessive breakdown of extracellular matrix (ECM) proteins. Uncontrolled ECM degradation can lead to severe tissue damage and contribute to tumor invasion, metastasis, and inflammation in various types of cancer [71,72]. Natural compounds from wine could control the activity and expression of MMP-2 and MMP-9; regarding this insight, we choose flavonoid and non-flavonoid compounds to control their functions.

### 3.1. Flavonoids

Flavonoids are essential naturally occurring polyphenolic compounds with a characteristic structure consisting of a heterocyclic oxygen-containing ring and two phenyl groups [73,74]. These bioactive compounds are widely distributed in flowers, vegetables, fruits, seeds, and wines [75]. Flavonoids exhibit diverse biological activities, including hormonal regulation, cardioprotective effects, and anticancer properties [75,76]. Structurally, flavonoids are classified into six groups: flavonol, flavanol, flavone, flavanone, isoflavone, and anthocyanidin [77].

Among these, multiple flavonols in wine have been reported as regulators of MMP-2 and MMP-9, enzymes involved in cancer progression. **Quercetin** (**Que**) is a flavonoid abundant in grapes, foliage, fruits, vegetables, and red wine [72]. It possesses diverse pharmacological properties, including anti-viral, anti-bacterial, anti-cancer, immune-modulatory, and radical-scavenging activities [78]. Studies have shown that **Que** inhibits cancer progression by promoting apoptosis, inducing cell cycle arrest, suppressing angiogenesis, and blocking metastasis via multiple intracellular signaling pathways, including MAPK, p53, Wnt/β-catenin, PI3K/Akt, and NF-κB [79,80]. Tang et al. reported that **Que** treatment increased the expression of TIMP-1 and TIMP-2 in a concentration-dependent manner while significantly reducing MMP-2 and MMP-9 activity and expression in breast cancer cells. Similarly, in pancreatic cancer, **Que** at concentrations of 20, 40, and 80 μM inhibited MMP-2 expression, as determined by gelatin zymography [64,81]. Furthermore, **Que** has also been shown to suppress breast cancer cell adhesion, invasion, and migration [82].

Another flavonol, **kaempferol** (**Kae**), is naturally present in grapes, blackberries, tea leaves, and broccoli [83]. It possesses potent anti-oxidant, anti-cancer, anti-viral, and anti-inflammatory properties [84,85,86]. **Kae** has been demonstrated to inhibit VEGF-mediated angiogenesis in OVCAR-3 and A2780 cells and suppresses MMP-2 activity by activating AP-1 and blocking ERK1/2 phosphorylation [87]. Additionally, **Kae** reduces cell invasion by inhibiting AKT phosphorylation without interfering with the MAPK pathway, thereby decreasing MMP-9 activity [83,88]. Gelatin zymography further confirmed that **Kae** at 50 μM significantly decreased MMP-2 and MMP-9 activity in SK-Hep-1 and Huh-7 liver cancer cells [83,84,85,86,87,88,89].

Similarly, **myricetin** (**Myr**) is another flavonol commonly found as a glycoside form (O-glycosides) in nuts, berries, fruits, vegetables, herbs, and alcoholic beverages with anti-inflammatory and anti-oxidant properties [90,91,92]. Western blot and gelatin zymography analyses revealed that **Myr** treatment effectively suppressed MMP-2 and MMP-9 activity in MDA-Mb-231Br breast cancer cells summarized in Table 2. After 24 h exposure to **Myr** at 5 and 10 μM, a decrease in the levels of MMP-2 (ca. 30%) and MMP-9 (ca. 50%) was observed. Additionally, **Myr** significantly downregulated mRNA expression of MMP-2 and MMP-9 in a dose-dependent manner [90,91,92,93].

**Luteolin** (**Lut**) is a flavonoid belonging to the flavone subclass, commonly found in tea, fruits, and vegetables. It is known for its anti-inflammatory, anti-allergic, anti-mutagenic, and anti-oxidant properties [94,95,96,97]. **Lut** has been shown to inhibit PI3K/Akt signaling, which is essential for MMP-2 and MMP-9 expression. In an A375 melanoma model, **Lut** treatment significantly reduced tumor weight and suppressed MMP-2 and MMP-9 expression, demonstrating its strong anti-cancer potential [98].

**Apigenin** (**Api**) is a flavone derivative, found in fruits, vegetables, and wine [99]. **Api** has some health benefits, including anti-oxidant, anti-inflammatory, anti-depressive, anti-bacterial, anti-viral, and anti-cancer activities [100,101]. AKT phosphorylation and p-p70S6K1 expression were significantly reduced by **Api** administration. As a result, MMP-9 and p-AKT expression levels were considerably lower in OVCAR-3 cells, which are listed in Table 3 [102].

Flavanols in wine, such as **epicatechin** (**EC**) and **epigallocatechin gallate** (**EGCG**), are flavonoids with notable anti-cancer effects via regulation of oxidative stress, inflammation, and MMP activity. Specifically**, EC** is a non-toxic compound present in tea, onions, beans, citrus fruits, and grapes. In addition to its potent anti-cancer properties, **EC** plays a role in regulating reactive oxygen species (ROS) formation and inflammation [103,104,105,106]. In H1299 and A549 cells, **EC** reduced MMP-9 activity by renovating DNA damages, leading to decreased migration and invasion while promoting apoptosis and enhancing radiosensitivity [107].

**EGCG**, a phenolic compound mostly found in grape seeds, contributes to the taste and astringency of wine and grape juice [108]. In MDA-MB-231 cells, **EGCG** can control different cancers in the mouth, small intestine, liver, pancreas, throat, colon, prostate, lung, stomach, and skin [109]. Zymography analysis revealed that the activity of MMP-2 and MMP-9 were decreased upon treatment of **EGCG** in mouse lung carcinoma cells, reinforcing its potential as an anti-cancer agent as shown in Table 4 [110,111].

**Astilbin** (**Ast**) is found in wine and medicinal herbs like the rhizome of *Smilax china* L. [112]. **Ast** has shown various activities including anti-inflammatory and immunoregulatory effects [113,114]. **Ast** administration was shown to lower TGF-β1 and CTGF levels and Western blot revealed that **Ast** therapy mitigated high glucose-induced decreases in MMP-2 and MMP-9 expression in HBZY-1 cells, as shown in Table 5 [115]. A dihydroflavonol **taxifolin** (**Tax**) is present in milk thistle, citrus fruits, vinegar, olive oil, and wines, possessing multiple biological functions, including anti-oxidant, anti-bacterial, anti-carcinogenic, metal-binding, and ROS scavenging [116,117]. In gastric cancer cells (AGS and NCI-N87), **Tax** inhibited colony formation and wound healing, leading to reduced MMP-2 and MMP-9 expression. Additionally, **Tax** treatment increased ZO-1 and E-cadherin levels in AGS and NCI-N87 cells while decreasing *N*-cadherin, suggesting inhibition of epithelial-mesenchymal transition (EMT) and cancer metastasis [118]. **Genistein** (**Gen**), a mixture of isoflavone and phytoestrogen abundance in grapes and wine, has been linked to reduced prostate cancer risk in men and altered breast cancer risk in Asian women [119,120]. **Gen** exerts its anti-cancer effects by inhibiting NF-κB activation, preventing its nuclear translocation, and thereby suppressing MMP-2 and MMP-9 activity in mouse cancer models presented in Table 5 [121,122].

**Naringenin** (**Nar**), a flavanone found in tomatoes, citrus fruits, and wine, exerts strong anti-oxidant effects due to its hydroxyl group at the C-5 position [123]. Western blot and gelatin zymography analyses revealed that **Nar** treatment reduced botjMMP-2 and MMP-9 protein levels and enzymatic activity in U87 cells [124]. TGF-β assists MMP-2 and MMP-9 promote tumor growth and metastasis. On the other side, **Nar** (20–160 μM) has been shown to suppress MDA-MB-231 breast cancer proliferation [125,126]. In vitro studies demonstrated that **Nar** (100 and 200 μM for 48 h) significantly reduced MMP-2 and MMP-9 expression in A549 cells, exhibiting anti-proliferative and anti-metastatic effects [127]. Additionally, **Nar** (100, 200, and 300 μM) inhibited p38 and ERK signaling pathways, leading to reduced MMP-2 and MMP-9 activity [128]. Similarly, **Nar** (20, 40, and 80 μM) downregulated MMP-2 and MMP-9 expression in SGC-7901 gastric cancer cells, further supporting its metastasis-inhibitory potential [129,130,131,132,133].

**Naringin** (**Nrg**), found in grapefruit, citrus plant species, and wine, possesses a broad range of therapeutic properties, such as anti-inflammatory, anti-oxidant, anti-cancer, and cognitive activities [134]. **Nrg** has also been demonstrated to suppress the occurrence of human tumors by inhibiting angiogenesis [135]. Western blot and gelatin zymography analysis were employed for figuring out the expression and activity of MMP-2 and MMP-9 in response to **Nrg** (5, 10, and 20 mM) concentrations in U87 cells. The analysis revealed that Nrg downregulated MMP-2 and MMP-9 expression by inhibiting p38 and MAPK phosphorylation [125].

**Hesperetin** (**Hsp**) is commonly found in grapes, oranges, and wine [136]. **Hsp** has medicinal advantages such as anti-allergic, anti-hyperglycemic, anti-hyperlipidemic, and anti-cancer properties [136]. **Hsp** reduced the development of tumors in MCF-7 cells by suppressing Bcl-xL in MMP-2 and MMP-9 [137,138]. Researchers reported that **Hsp** suppressed MMP-9 expression at concentrations of 95 μM in MCF-7 cells and 50–100 μM in 4T1 cells, respectively [139,140]. **Xanthohumol** (**Xn**), a phenolic compound rich in hops, wine, and beer, could inhibit cancer progression by blocking MMP-2 and MMP-9 activity [141,142,143,144,145,146]. In A549 lung cancer cells, **Xn** (10 μM) significantly suppressed MMP-9 expression, as confirmed by Western blot analysis [146]. An isomer of **Xn**, **isoxanthohumol** (**Ixn**), could modulate NF-κB, Akt, and ERK signaling pathways, further inhibiting MMP-2 and MMP-9 expression in MDA-MB-231 cells, as briefly summarized in Table 6 [147,148,149,150].

### 3.2. Non-Flavonoids

The non-flavonoid phenolic and non-phenolic compounds in wine are divided into hydroxybenzoic acids, hydroxycinnamic acids, stilbenes, and miscellaneous compounds (e.g., coumarins, and ellagic acid). These compounds not only contribute to stabilizing the color of red wine through intra- and intermolecular reactions but also play a significant role in enhancing the wine’s flavor. Furthermore, some of them exhibit potent anti-oxidant and anti-cancer activities [151].

**Gallic acid (GA)** is a polyhydroxy phenolic molecule found in many sources such as fruits, vegetables, and other foods [152]. It has demonstrated a range of biological properties, such as anti-cancer, anti-inflammatory, and anti-bacterial properties [152,153]. **GA** has been shown to regulate MMP-2 and MMP-9 activity in K562 cells, with the hydroxyl group at the para-position of the carboxylic group being essential for this effect. Furthermore, **GA** could induce JNK inactivation in BAPTA-AM (Ca^2+^ chelator) and diminish the activity of MMP-2 and MMP-9 with Ca^2+^ playing a significant role in this process [154,155]. In addition, a study by Liu et al. discovered that **CA** increases the levels of the inhibitor TIMP-1, which leads to a significant reduction in MMP-2 and MMP-9 activity in prostate cancer cells (PC-3) [156].

The natural polyphenol **ellagic acid** (**EA**), present in grapes, strawberries, and nuts, has a greater concentration in red wine than resveratrol [156]. **EA** is well-known for its anti-oxidant and cancer-preventive properties [157,158,159,160,161]. **EA** demonstrates anti-cancer properties by stopping the cell cycle and cell growth, triggering cell death and reducing inflammation both in vitro and in vivo [162,163,164,165]. Western blot analysis by Huidi Liu et al. evaluated the effects of EA (10–15 mg/mL) on MMP expression in A2780 cells. The results showed that after a 24 h treatment, **EA** significantly suppresses the proliferation, migration, and progression of cancer by down-regulating the expression of MMP-2 and MMP-9, as shortly described in Table 7 [166].

**Caffeic acid** (**CA**) is a mixture of hydroxycinnamate and phenylpropanoid found in coffee, tea, wine, blueberries, apples, cider, and honey. **CA** may possess anti-bacterial, anti-diabetic, anti-oxidant, cardioprotective, and anti-inflammatory effects [167]. One of its mechanisms of action involves reducing vascularization through the inhibition of VEGF, which leads to decreased tumor growth [168]. Due to NF-κB stimulation in tumor cells, MMP-2 and MMP-9 break down ECM type IV collagen during cancer invasion and metastasis [169]. A recent study has shown that **CA**’s inhibitory effects on MMP-2 and MMP-9 are linked to its ability to block NF-κB activation, thereby reducing tumor development and spread in hepatocellular carcinoma cancer cells (HCC) [152].

Fruits (grapes, apples, and pears), vegetables (tomatoes, carrots, garlic, onions, and potatoes), mushrooms, and drinks (teas, coffee, and wines) are also abundant sources of ***p*-coumaric acid** [170,171,172,173,174]. ***p*-coumaric acid** has garnered a lot of interest because of its anti-cancer properties [170,175]. According to a study by S. Pragasam et al., ***p*-coumaric acid** suppresses the NF-κB and TNF-α genes’ activity, which are involved in inflammation and cancer progression [176]. Furthermore, ***p*-coumaric acid** inhibits NF-κB nuclear translocation by preventing the phosphorylation of its p65 subunit, thereby reducing COX-2 production [177]. It also directly binds to MMP-9, which further impedes NF-κB nuclear migration and suppresses MMP-9 gene expression in mice, as concisely represented in Table 8 [178].

**Piceatannol** (**Pic**) is a phenolic molecule abundant in grapes and red wine. **Pic** has anti-inflammatory and anti-cancer effects via downregulating NF-κB [179]. Specifically, the MMP gene promoter is regulated by NF-κB [180]. Previous studies have demonstrated that **Pic** inhibits the translocation of p65 and p50 proteins, thus regulating NF-κB activity. According to the current research, **Pic** downregulates the activity of NF-κB and enhances anti-tumor properties by suppressing the expression of MMP-9 in HPC cells [179,180,181]. Moreover, **Pic** directly inhibited PI3K activity, which decreased H-ras-induced Akt phosphorylation. The PI3K/Akt pathway plays a role in both the invasion and migratory capacity of H-ras MCF10A cells and the activation of MMP-2 activity [182]. Thus, the inhibition of PI3K by **Pic** not only suppresses Akt activation but also reduces MMP-2 activity, thereby impairing the invasive and migratory capabilities of the cells [183].

**Resveratrol** (**Rsv**) is a phenolic organic compound rich in peanuts, berries, and grapes. Wine and other alcoholic beverages are the major sources of **Rsv** [184]. **Rsv** has broad activities, such as anti-viral, anti-bacterial, anti-fungal, anti-inflammatory, anti-aging, and anti-oxidant properties [185]. Gelatin zymography reveals that **Rsv** significantly decreases MMP-2 and MMP-9 activity in a dose and time-dependent manner. Specifically, a zymography study has shown that **Rsv** suppresses MMP-2 and MMP-9 expression in HTB94 cells [186]. Additionally, **Rsv** also reduced human lung adenocarcinoma cell metastasis by suppressing HO-1. HO-1 inhibition or silencing NF-κB signaling indicates **Rsv** acts as an MMP-2 and MMP-9 inhibitor, as shown in Table 9 [187].

Wine also contains various essential nutrients, including riboflavin (vitamin B2) and folic acid (vitamin B9), which play important roles in cellular processes and metabolism. **Riboflavin** (**RF**) is a component of the vitamin B2 complex [188]. It was discovered that milk, eggs, lean meats, green leafy vegetables, and wine contain a substantial amount of **RF** [29]. **RF** supports vital physiological processes such as cell division and energy metabolism, and also reduces ROS and mRNA expression of MMP-2 and MMP-9 [189,190]. Gelatin zymography data demonstrate that iRF (30, 50 mM) suppresses MMP-2 and MMP-9 activity and expression in B16F10 cells. This result shows that **RF** has enormous potential as an anti-cancer agent [191].

**Folic acid** (**FA**) is well known by its alternative name, vitamin B9. Vegetables, fruits, legume seeds, cereal grains, wheat germs, peas, soybeans, beans, and wine are major sources of **FA** [192,193,194]. A recent study found that FA downregulates MMP-9 activity, particularly during the early stages of spinal cord injury (SCI), when MMP-9 is highly expressed. In male Sprague Dawley rats, FA treatment reduces cSCI-induced NP by suppressing the expression of MMP-2 and MMP-9, shown in Table 10 [193,194].

Although numerous in vitro studies have demonstrated the inhibitory effects of wine-derived compounds on MMP-2 and MMP-9 expression in tumor cells, translating these findings into in vivo contexts remains challenging. A major limitation is the poor bioavailability of many polyphenolic compounds and other constituents found in wine, which may hinder their efficacy in reducing MMP expression in tumors within a physiological setting [195]. For instance, resveratrol, quercetin, and catechins are known to be rapidly metabolized and poorly absorbed, resulting in insufficient plasma and tissue concentrations to exert meaningful biological effects [196,197]. Therefore, further in vivo investigations are important to determine whether these compounds can reach sufficient concentrations at tumor sites to exert meaningful biological effects, particularly in modulating MMP-2 and MMP-9 expression and thereby inhibiting tumor progression and metastasis.

## 4. Conclusions

This review provides a focused synthesis of current knowledge regarding the regulatory effects of wine-derived bioactive compounds on MMP-2 and MMP-9 in the context of cancer progression. These compounds exert their effects by modulating critical signaling pathways, including MAPK, PI3K/Akt, and NF-κB, leading to the downregulation of MMP expression and activity, then the attenuation of tumor invasiveness and metastasis. While many in vitro studies have demonstrated promising anti-metastatic and anti-invasive effects across various cancer cell lines, in vivo data remain limited and often complicated by the low bioavailability of these compounds. This is the first comprehensive effort to systematize and critically evaluate the relationship between wine-derived secondary metabolites and MMP regulation in cancer. Further mechanistic studies, pharmacokinetic evaluations, and translational research, including in vivo validation and clinical exploration, are necessary to determine the true therapeutic potential of these compounds. Ultimately, these bioactive agents may represent valuable adjuncts to existing cancer therapies, especially in targeting tumor invasiveness and metastasis via MMP suppression.

## Figures and Tables

**Table 1:** Bioactive compounds and their concentrations in wine.

	Bioactive Compounds	Amounts in Wine (mg/L)	Refs.
Flavonoids	Quercetin	8.3	[9,20]
	Kaempferol	2.3	[9]
	Myricetin	8.3	[9]
	Luteolin	0.2–7.2	[21,22]
	Apigenin	0.2	[21,23]
	Epicatechin	3.3	[24]
	Epigallocatechin gallate	5–20	[9]
	Taxifolin	0.65–9.6	[21,25]
	Genistein	0.01	[9,26]
	Naringenin	0.1–19.8	[21,23]
	Naringin	7.5	[9]
	Hesperetin	0.5	[9]
	Xanthohumol	0.002–1.2	[9]
	Isoxanthohumol	0.04–3.4	[9]
Non-flavonoids	Gallic acid	2–130	[21,27]
	Ellagic acid	8.9	[24]
	Caffeic acid	0.3–26	[21,27]
	*p*-coumaric acid	0.4–15	[9]
	Piceatannol	5.8	[9]
	Resveratrol	0.5–7	[21,28]
	Riboflavin	0.0085–0.1349	[29,30]
	Folic acid	0.0004–0.0045	[29,30,31,32]

**Table 2:** Flavonols in wine and their influence on the activity or expression of MMP-2 and MMP-9.

Compounds	MMP-2 Activity/Expression	MMP-9 Activity/Expression	Cell Lines/ Animals	Refs.
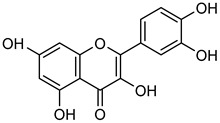 **Quercetin**	Down regulation (↓/↓)	Down regulation (↓/↓)	MDA-MB-231 cells	[64,81,82]
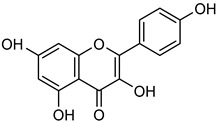 **Kaempferol**	Down regulation (↓/-)	Down regulation (↓/-)	A2780 cells OVCAR-3 cells SK-Hep-1 cells Huh-7 cells	[83,84,85,86,87,88,89]
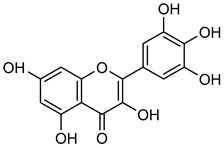 **Myricetin**	Down regulation (↓/↓)	Down regulation (↓/↓)	MDA-Mb-231Br cells	[90,91,92,93]

**Table 3:** Activity or expression of flavones in MMP-2 and MMP-9.

Compounds	MMP-2 Activity/Expression	MMP-9 Activity/Expression	Cell Lines/Animals	Refs.
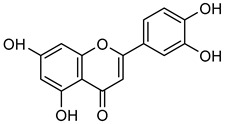 **Luteolin**	Down regulation (-/↓)	Down regulation (-/↓)	A375 cells	[98]
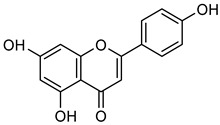 **Apigenin**	Not applicable	Down regulation (-/↓)	OVCAR-3 cells	[102]

**Table 4:** Summary of the activity or expression of MMP-2 and MMP-9 by flavanols.

Compounds	MMP-2 Activity/Expression	MMP-9 Activity/Expression	Cell Lines/ Animals	Refs.
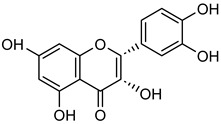 **Epicatechin**	Not applicable	Down regulation (↓/-)	H1299 cells A549 cells	[106,107]
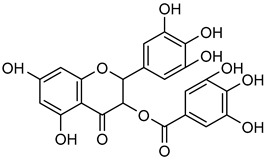 **EGCG**	Down regulation (↓/-)	Down regulation (↓/-)	OVCAR-3 cells	[110,111]

**Table 5:** Flavanonols and isoflavone in wine and their influence on the activity or expression of MMP-2 and MMP-9.

Compounds	MMP-2 Activity/Expression	MMP-9 Activity/Expression	Cell Lines/Animals	Refs.
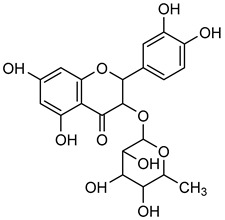 **Astilbin**	Down regulation (-/↓)	Down regulation (-/↓)	HBZY-1 cells	[115]
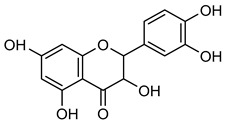 **Taxifolin**	Down regulation (-/↓)	Down regulation (-/↓)	NCI-N87 cells AGS cells	[118]
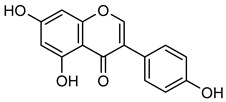 **Genistein**	Down regulation (↓/-)	Down regulation (↓/-)	MDA-Mb-231Br cells	[121,122]

**Table 6:** Brief description of flavanone activity or expression of MMP-2 and MMP-9.

Compounds	MMP-2 Activity/Expression	MMP-9 Activity/Expression	Cell Lines/ Animals	Refs.
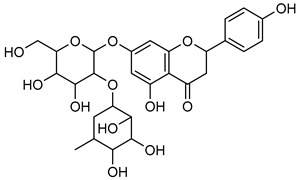 **Naringenin**	Down regulation (↓/↓)	Down regulation (↓/↓)	MDA-MB-231 cells SGC-7901 cells A549 cells U87 cells	[124,125,126,127,128,129,130,131,132,133]
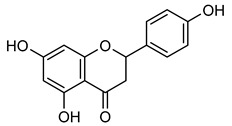 **Naringin**	Down regulation (↓/↓)	Down regulation (↓/↓)	U87 cells	[125]
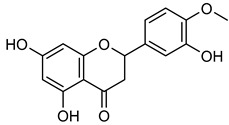 **Hesperctin**	Down regulation (↓/↓)	Down regulation (↓/↓)	MCF-7 cells 4T1 cells	[137,138,139,140]
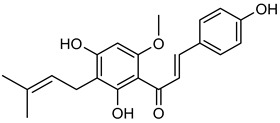 **Xanthohumol**	Down regulation (↓/-)	Down regulation (↓/↓)	A549 cells	[146]
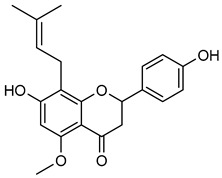 **Isoxanthohumo**	Down regulation (-/↓)	Down regulation (-/↓)	MDA-MB-231 cells MonoMac6 cells	[147,150]

**Table 7:** Hydroxybenzoic acids in wine and their influence on the activity or expression of MMP-2 and MMP-9.

Compounds	MMP-2 Activity/Expression	MMP-9 Activity/Expression	Cell Lines/ Animals	Refs.
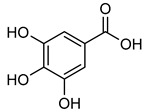 **Gallic acid**	Down regulation (↓/-)	Down regulation (↓/-)	K562 cells PC-3 cells	[154,155,156]
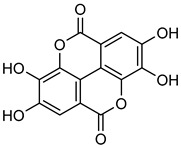 **Ellagic acid**	Down regulation (-/↓)	Down regulation (-/↓)	A2780 cells	[166]

**Table 8:** Hydroxycinnamic acids in wine and their influence on the activity or expression of MMP-2 and MMP-9.

Compounds	MMP-2 Activity/Expression	MMP-9 Activity/Expression	Cell Lines/ Animals	Ref.
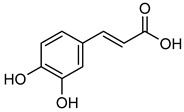 **Caffeic acid**	Down regulation (↓/-)	Down regulation (↓/-)	HCC cells	[152]
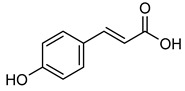 ***p*-coumaric acid**	Not Applicable	Down regulation (-/↓)	Mice	[178]

**Table 9:** Impacts of stilbenes on the activity or expression of MMP-2 and MMP-9.

Compounds	MMP-2 Activity/Expression	MMP-9 Activity/Expression	Cell Lines/ Animals	Refs.
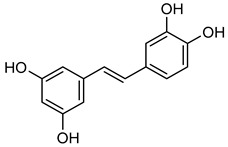 **Piceatannol**	Down regulation (↓/-)	Down regulation (↓/-)	MCF10A cells HPC cells	[179,180,181,182,183]
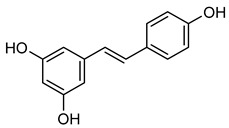 **Resveratrol**	Down regulation (↓/↓)	Down regulation (↓/↓)	HTB94 cells	[186,187]

**Table 10:** Vitamins in wine and their influence on the activity or expression of MMP-2 and MMP-9.

Compounds	MMP-2 Activity/Expression	MMP-9 Activity/Expression	Cell Lines/ Animals	Refs.
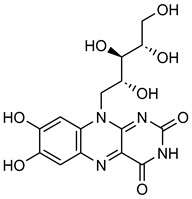 **Riboflavin**	Down regulation (↓/↓)	Down regulation (↓/↓)	MCF10A cells	[191]
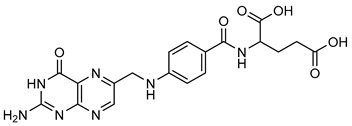 **Folic Acid**	Down regulation (-/↓)	Down regulation (↓/↓)	Sprague Dawley Rats	[193,194]

## Data Availability

Not applicable.

## Abbreviations

The following abbreviations are used in this manuscript:

MMP	Matrix metalloproteinase
HDL	High-density lipoprotein
ECM	Extracellular matrix
GSE	Grape seed extract
MAPK	Mitogen-activated protein kinase
ERK	Extracellular signal-regulated kinase
CoA	Coenzyme A
NF-κB	Nuclear factor-kappa B
NSCLC	Non-small cell lung cancer
COX-2	Cyclooxygenase-2
TNF-α	Tumor necrosis factor
TIMP-1	Tissue inhibitor of metalloproteinase-1
TIMP-2	Tissue inhibitor of metalloproteinase-2
VEGF	Vascular endothelial growth factor
SCI	Spinal cord injury
